# Evaluation of a web-based back prevention program for primary school children: a randomized controlled trial

**DOI:** 10.1038/s41598-025-27813-0

**Published:** 2025-11-21

**Authors:** Samuel Weigel, Joachim Grifka, Petra Jansen

**Affiliations:** 1 Department of Sports Sciences, University of Regensburg, Regensburg, Germany; 2 Head of Orthopaedics und Ergonomics, Regensburg University of Applied Sciences, Regensburg, Germany

**Keywords:** Back pain prevention, Primary-school children, Posture, Psychological well-being, Trunk muscle endurance, Functional mobility, Paediatric research, Orthopaedics

## Abstract

Back pain and postural abnormalities are increasingly prevalent among primary school children and may persist into adulthood. This randomized controlled trial evaluated a 12-week web-based preventive back-care intervention for 141 children aged 6–11 years. The intervention group (*n* = 71) received exercise and back-oriented education videos, while the control group (*n* = 70) received only educational videos regarding general health promotion. Outcomes included short-term goals such as postural evaluation, back pain, and postural endurance, as well as prevention-oriented outcomes including trunk endurance, functional mobility, back-related knowledge, and psychological well-being (self-compassion and self-concept), complemented by parent-reported information. Exploratory analyses examined subgroup differences in program response. The intervention significantly increased back-related knowledge (*p* < .001, partial η² = 0.228) and positive self-compassion (*p* = .011, partial η² = 0.045), but did not lead to significant group differences in posture, back pain prevalence, trunk muscle endurance, functional mobility, self-concept, negative self-compassion, or daily sitting time. Adherence varied, and postural assessment was limited by the lack of a standard medical examination, which should be considered when interpreting the results. These findings highlight challenges in digital health promotion for young children and suggest that future programs may benefit from increased interactivity, parental involvement, and in-person components to effectively support spinal health in this age group.

**Supplementary Information:**

The online version contains supplementary material available at 10.1038/s41598-025-27813-0.

## Introduction

### Postural health in childhood

Back pain in children and adolescents is said to increase with age and also become more prevalent over time. Data from the second wave of the nationwide study on the health of children and adolescents in Germany (KiGGS) confirm this trend, showing a rising prevalence of back pain among children and adolescents aged 3 to 17 years^1^. In adulthood, back pain remains widespread and is one of the most common health complaints^2^.

In addition to pain, postural deviations are frequently observed in children^3–6^. Studies indicate that children who do not participate in sports are more likely to exhibit postural abnormalities than physically active ones^4^. However, the relationship between posture and back pain is complex and insufficiently understood^7^. While some studies have found no direct association^8^, others have identified a link between head position and neck pain in adults^9^. Similarly, a study among Czech schoolchildren found that those with poorer posture reported back pain more frequently than their peers with normal posture^4^.

### The need for innovative early intervention

Because adolescent spinal pain has been identified as a predictor of spinal pain in adulthood^10^, early prevention programs are needed, as also highlighted by Yang et al.^6^. Preventive interventions may be particularly important when movement patterns and postural habits are established^11^ during primary school age. Declining physical activity and increasing sedentary behavior, partly due to technological change, further contribute to early risk factors for spinal health issues^12,13^.

Despite the topic’s growing relevance, research on the effectiveness of back pain prevention programs in children remains limited compared to the adult population, underscoring the need for further investigation^7,14^. Most studies in children focus on physical aspects such as posture and muscular endurance, while fewer address psychological components like well-being or self-compassion^15^. Given the influence of psychosocial factors on back pain^7^, a more holistic approach is needed in preventive strategies for children.

Program adherence and the development of sustainable habits are key determinants of effectiveness in child-focused back prevention interventions^16^. However, Hill & Keating^16^ highlight that long-term engagement is particularly challenging in younger populations and emphasize the need for more engaging approaches. To enable meaningful participation, the program content and the assessment procedures must be tailored to children’s developmental stage, age, and cognitive abilities^17^.

Taken together, these findings underscore the need for innovative and accessible preventive strategies to promote spinal health in children. Given these challenges and the need for age-appropriate approaches, digital health promotion has emerged as a promising strategy to deliver effective interventions in this population.

### The potential of digital health promotion

The importance of digital health promotion is growing as a key strategy for engaging young people in preventive and health-promoting interventions^18^. It offers several advantages, including broad accessibility, cost-effectiveness, and flexible personalization. Moreover, digital formats can foster social connections, which may serve as an additional source of motivation. However, it is essential that such programs are evidence-based and their effectiveness systematically evaluated^19^. A further strength of digital health interventions is the opportunity to gamify health-related habits and content, which has been shown to increase engagement and adherence, particularly among younger populations^20^. Moreover, the integration of artificial intelligence into exercise and health promotion programs is gaining attention, with recent reviews highlighting its potential to personalize, monitor, and enhance physical activity interventions across age groups, including children^21^. Adolescents and young adults are the primary users of digital health applications and are generally familiar with technological devices^22^. While most research has focused on these age groups, digital interventions have also been applied in younger populations. They demonstrated success in areas such as reducing sedentary behavior, increasing physical activity^23^, and supporting the prevention and treatment of childhood overweight, including nutrition^20,24^. However, despite the growing prevalence of back-related issues in children, digital interventions specifically targeting spinal health in primary school-aged children remain scarce. This age group is particularly important, as it represents a developmental stage in which postural habits and physical activity patterns are established, potentially influencing long-term spinal health. Addressing this gap is critical to determine whether digital approaches can support sustainable engagement, promote health-related behavioral change, and positively impact spinal health outcomes in this young population^19^ To translate this potential into effective practice, it is important to identify which specific physical, psychological, and behavioral outcomes are most relevant for promoting spinal health in primary school-aged children. The following section outlines the targeted outcomes that informed the design and evaluation of the BackFit program.

### Target outcomes of a multidimensional preventive back intervention

Research has shown that postural anomalies are associated with trunk strength, suggesting that targeted core exercises may contribute to postural improvements^25^. Postural deviations have also been linked to weakened spinal muscles^26^, which may serve as predictors for the development of low back pain^27^. Furthermore, exercise interventions have been shown to reduce lower back pain in children^28^. Three of the four studies included in the review used combined approaches incorporating strength, flexibility, coordination, and aerobic exercises, with some using functional exercises and some delivered under the supervision of a physiotherapist. The other intervention examined in the review used a foam seat wedge to tilt the school seating surface forward by 10 degrees. A more recent review has also identified Pilates, as a core-oriented exercise approach, to be effective in reducing low back pain. This type of training demonstrated advantages both compared to no exercise and to non-specific exercise programs^29^. A review by Kamper et al.^7^ found that prevention programs combining exercise and education are more effective in reducing pain than home exercise alone or no intervention. However, the overall quality of evidence remains limited, primarily due to the small number of treatment studies in young populations.

Back-care interventions that include educational content have also been effective in improving back-related knowledge among school-aged children^30^. In addition, studies indicate that core conditioning programs can enhance trunk muscle endurance^31^. Functional mobility has been positively linked to both physical activity and core strength^32,33^, emphasizing the potential benefits of structured exercise programs targeting these areas.

Beyond physical aspects, body awareness has been linked to increased levels of physical activity^34^, and embodiment theory suggests that improvements in posture and bodily awareness can positively influence psychological well-being^35^. Research further supports the connection between physical activity and mental well-being in children^36^. Although interventions aimed at reducing sedentary behavior and increasing physical activity have been shown to improve psychological well-being in children and adolescents, the effects appear more pronounced in adolescents^37^.

The BackFit program was conceptualized as a preventive intervention aiming to promote spinal health in primary school children while also achieving short-term improvements in posture, back pain, and health-related behaviors through a structured, engaging digital format. Given that the BackFit program is designed to increase physical activity, provide targeted training for the trunk muscles using approaches similar to those found effective in RCTs, raise awareness of the importance of posture, and enhance back-related knowledge through conscious engagement with these topics, we expected corresponding improvements in these areas among participants. Based on these considerations, the following main hypotheses were proposed:

#### H1

The intervention group will significantly reduce postural anomalies and reported back pain more than the control group.

#### H2

The intervention group will improve trunk muscle endurance and functional mobility more than the control group.

#### H3

Compared to the control group, the intervention group will show more significant improvements in psychological well-being, specifically in self-compassion and self-concept.

#### H4

The intervention group will increase back-related knowledge more than the control group.

The four hypotheses represent the predefined primary outcome domains of the study. In addition to the analysis of these main outcomes, additional exploratory analyses were conducted; no directed hypotheses were formulated for these comparisons. As Dullien et al.^30^ found that the ability to maintain an upright posture improved equally in both experimental and control groups over a period of 10 months among German fifth-grade students, we aimed to explore potential changes in postural endurance in both groups. We also explored changes in parent-reported daily sitting times to examine whether the intervention leads to a reduction in sedentary behavior. In addition, we investigated the potential influence of the demographic factors of sex, and socioeconomic status on the observed changes in an exploratory manner. Furthermore, exploratory subgroup analyses within the intervention group were conducted to identify potential factors associated with intervention responsiveness. Specifically, we compared changes in motor performance between three age cohorts, examined potential differences by sex, SES, and adherence and explored whether recent growth or baseline performance predicted improvements. Aiming at these items, a principal limitation is that no usual medical examination of the status of back muscles status and posture was performed.

## Materials & methods

### Participants

Between January 2023 and November 2024, participants were recruited through pediatric practices, youth organizations, religious communities, sports clubs, the university’s family support services, and local media outlets. The project was also promoted via local radio broadcasts. A power analysis^38^ (d = 0.25, β = 0.80, α = 0.0125) indicated that a sample size of 182 children was required. To account for an anticipated dropout rate of approximately 10%, the initial target was increased to 200 participants. However, this preregistered target could not be achieved due to recruitment challenges during a limited 1.5-year recruitment period and a fixed project timeline. Recruitment concluded with a total of 177 children, with 89 allocated to the intervention group (IG) and 88 to the control group (CG).

**Fig. 1:**
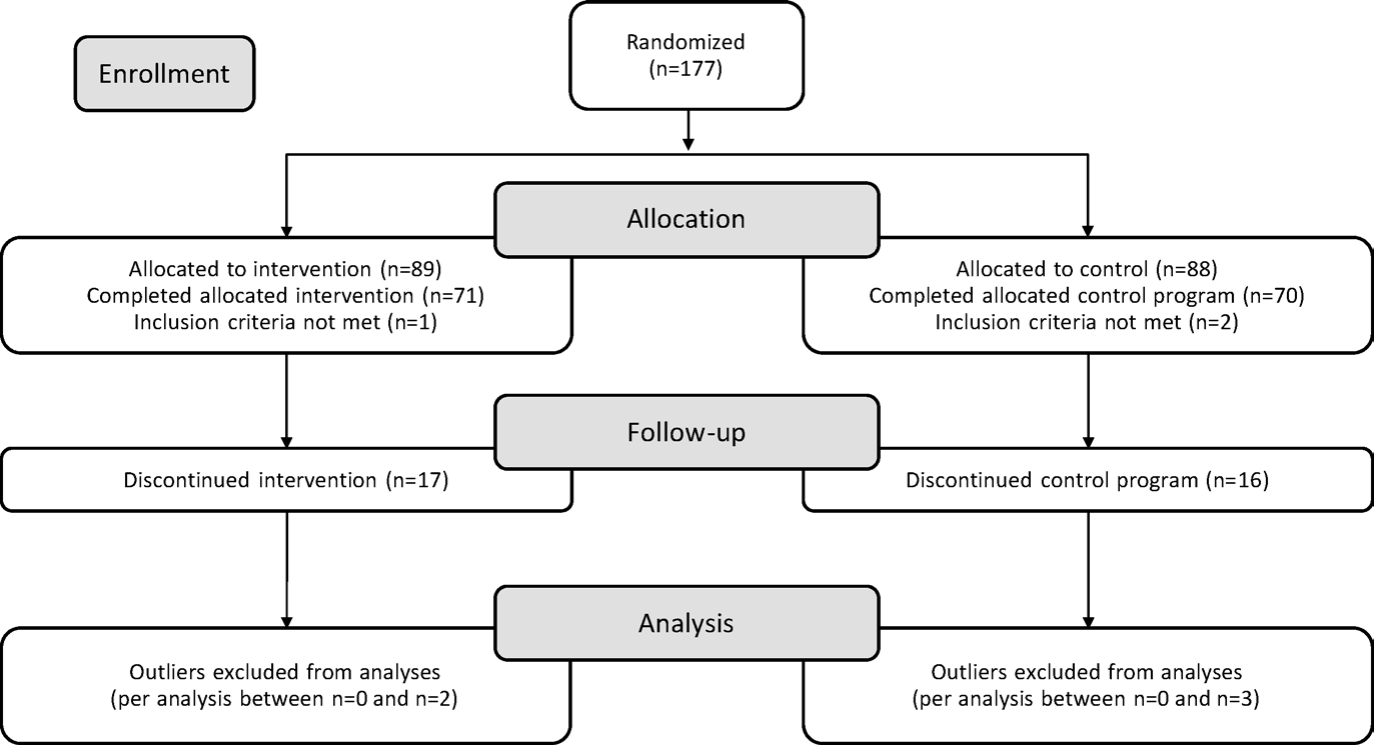
CONSORT 2010 flowchart of patient enrollment and randomization.

Exclusion criteria were as follows: (a) pre-existing structural conditions such as scoliosis, (b) medical restrictions preventing participation in strengthening exercises, (c) neurological disorders, (d) concurrent participation in other scientific intervention studies, (e) more than 10% missing responses in any administered questionnaire, (f) early program withdrawal, and (g) repeated delays making completion of the program within 15 weeks impossible. Based on these criteria, 36 participants were excluded from the analysis: one due to a diagnosed scoliosis (IG), one due to significant language barriers that prevented standardized testing (CG), and one for failing to complete more than 90% of several questionnaires (CG). The remaining excluded participants (17 IG, 16 CG) either withdrew from the program or could not complete it within 15 weeks due to repeated delays, as illustrated in Fig. 1.

The final dataset comprised 141 children aged 6 to 11 years (M = 8.60, SD = 1.30). Although the intended age range was 7 to 11 years, one six-year-old was included, as the child was nearing their seventh birthday at the time of data collection. Of the total sample, 65 participants were girls (46.1%) and 76 were boys (53.9%). Group allocation was nearly balanced, with 71 children in the IG and 70 in the CG.

### The backfit program

The BackFit program was designed to run over 12 weeks, with distinct content tailored to each group. Weekly videos were unlocked sequentially, requiring participants to complete the prior week’s content before accessing the next module. Unlocking occurred after seven days or immediately upon task completion if delayed beyond one week. While this flexible approach could extend the overall duration, it ensured thorough engagement with the material. The intervention was delivered via a web-based platform accessible remotely, and content was adapted to the specific needs of each group. To enhance motivation, participants could collect trophies and virtual coins by completing various tasks. These coins could be used to play short games, each lasting about five minutes, or to customize a personal avatar, increasing engagement with the program. The gamification elements were intentionally designed to require minimal screen time and served as short motivational rewards following the completion of physical activity and educational tasks, ensuring they did not replace or reduce the time spent on physical activity within the BackFit program. The structure of the program and its reward system was based on the successful and widely used learning app “Anton,” which was extensively used during the COVID-19 pandemic and has been shown to increase motivation among primary school children. The app covers all school subjects and grade levels, making it a familiar and accessible tool for children across different ages^39,40^. Screenshots of the exercise and knowledge videos can be found in Supplementary Figure S1.

#### Intervention group

The IG received two weekly videos: a 30-minute exercise video and a 5-minute knowledge video. The exercise videos focused on strengthening trunk muscles and were designed to be engaging and age-appropriate. To increase accessibility and appeal, each video was embedded in a storyline or included everyday household items such as towels, tennis balls, or toilet paper rolls to perform the exercises easily at home and to integrate movement into their familiar environment. An adult, a mascot and a child demonstrated exercises to ensure easy imitation and understanding, using storytelling or additional materials to increase motivation and active participation during the sessions. The cartoon-style knowledge videos addressed educational topics related to the structure and function of the spine, sedentary behavior, proper lifting techniques, and general principles of back-care in a child-friendly and engaging manner.

To further encourage healthy habits, the program included two optional daily components that aimed to support sustainable behavior change through daily micro-habits. First, participants could use a movement diary on the BackFit website to record their daily physical activity and sedentary behavior, promoting self-monitoring and reflection even in younger children. Second, they were encouraged to integrate six simple daily habits into their routines, such as performing a wall push-up after washing hands, using strategically placed reminder cards in the home environment to prompt activities and establish healthy routines in everyday contexts. These combined strategies were designed to go beyond conventional digital interventions by fostering active participation, habit formation, and movement in a playful, low-threshold, and structured manner.

#### Control group

The CG received one cartoon-style knowledge video per week, each lasting approximately five minutes. These videos focused on general health-related topics such as sleep hygiene and nutrition but did not include any back-specific content. The topics were inspired by the Klasse2000 health promotion and prevention program implemented in German primary schools^41^.

### Measurements

#### Posture assessment

Before the posture assessment, participants’ weight and height were measured. Postural abnormalities were evaluated using a slightly modified version of a visual assessment form^5^. Modifications were necessary because, for ethical reasons, participants wore shorts and tight-fitting tops, which limited the visibility of certain anatomical landmarks. If a top was too loose, it was temporarily adjusted with clothespins to improve the visibility of body contours. Thus, only a visual inspection for noticeable deviations was possible, and a standard medical examination could not be conducted.

Participants were observed standing in a relaxed, upright position. Assessed features included shoulder tilt, plumb line deviations, leg alignment, foot positioning, and other visible irregularities. The sagittal view was used to evaluate the cervical, thoracic, and lumbar spine curvature while wearing the outer garments. Functional tests were incorporated within the postural assessment to capture potential functional limitations in addition to static posture. The functional tests included single-leg stance, toe and heel walking, gait analysis, spinal rotation, lateral flexion, spinal extension, and finger-to-floor distance. For all functional tests except for the finger-to-floor distance and lateral flexion, it was only noted whether the movement could be performed, without further grading. As the finger-to-floor distance and lateral flexion closely resemble functional mobility assessments, changes in these measures were not analyzed in the study. However, they were assessed for completeness and are reported descriptively. The finger to floor distance was measured while participants stood on a low stool, allowing the fingertips to extend below the level of the feet during maximal forward bending with knees fully extended.

#### Acute back pain assessment

Following the physical examination, participants were asked about acute back pain. The assessment was explained to the children in age-appropriate language, describing back pain as any uncomfortable or painful feeling in the back, and they were encouraged to indicate if they were experiencing such sensations. If back pain was reported, children were asked about its location (pointing to a figure with outlined back contours), its intensity (assessed using a visual analogue scale), and possible aggravating factors, such as specific movements or activities that increased their pain.

#### Postural endurance assessment

To evaluate the ability to maintain an upright posture over time for exploratory purposes, the Matthiass test was administered, a component of a German posture evaluation for children^42^ and a motor performance test battery for use in children^43^. Participants were instructed to stand upright and hold their arms extended horizontally in front of a vertical grid marked with 5 × 5 cm squares. The test measured the time (in seconds) participants could maintain the posture without deviating by more than one square. The test was terminated if posture shifted beyond one grid line, the participant stopped voluntarily, or the maximum test duration was reached. The maximum score was 120 s following the test manual allowing for greater differentiation compared to the classic Matthiass test, which typically considers a duration of 30 s^43^. While the predictive value of the 30-second version of the Matthiass test in diagnostic settings has been questioned^44^, we employed it as an exploratory measure of postural endurance and trunk control in a functional, everyday-like task context. As this test has subsequently been used in publications for the quick and easy assessment of trunk control and postural endurance within the context of motor performance testing in young samples^30,45^, it was deemed appropriate to complement our postural assessment.

#### Trunk muscle endurance assessment

Trunk muscle endurance was assessed using two adapted McGill endurance tests^46^. For trunk extensor endurance, participants lay prone on a bench with their legs secured by straps. The upper body extended horizontally beyond the bench with arms crossed over the chest, while a horizontal bar placed at the upper back served as a reference to monitor alignment. The position’s duration was recorded, with a maximum limit of 300 s. The test was terminated after three instances of the back losing contact with the bar.

For trunk flexor endurance, participants maintained a 50° sit-up position supported by a wedge, which was shifted 10 cm backward at the beginning of the test. Feet were strapped, hands rested across the chest, and both knees and hips were flexed at 90°. Participants were instructed to hold the position without leaning back onto the wedge, rounding the back forward, or using their hands for support. The maximum duration was again limited to 300 s.

#### Functional mobility assessment

Functional mobility was assessed using the Functional Movement Screen (FMS)^47,48^. It comprises 10 exercises or clearing exams: deep squat, hurdle step (left/right), inline lunge (left/right), shoulder mobility (left/right), shoulder clearing test (left/right), active straight leg raises (left/right), trunk stability push-up, extension clearing test, rotary stability (left/right), and flexion clearing test. Each exercise was scored on a scale from 0 to 3, with a maximum possible total score of 21. The lower score of the two sides was used for exercises performed bilaterally. Any occurrence of pain during the exercises or clearing tests resulted in an automatic score of zero.

#### Psychological well-being assessment

This study selected self-concept as an indicator of psychological well-being next to self-compassion. The self-concept subscale of the German version of the “Beck Youth Inventories – 2nd Edition” (BSCI-Y)^49^ was utilized to assess psychological well-being. This subscale comprises 20 items, each rated on a 4-point Likert scale ranging from “never” (0) to “always” (3). All item scores were summed up to determine the total score, with a maximum achievable score of 60.

#### Self-compassion assessment

Self-compassion was measured using a German adaptation of the self-compassion scale short form designed for children^50^. The adaptation process involved two translators with advanced English proficiency who independently translated the questionnaire forward and backward. The scale consists of 12 items divided into two subscales, with responses recorded on a 5-point Likert scale ranging from ‘never’ (1) to ‘always’ (5). The two subscales assess self-judgment and isolation (negative self-compassion), and self-kindness and acceptance (positive self-compassion). Negatively phrased items were reverse-coded to ensure consistent scoring. Subscale scores were calculated as the mean of the six items within each respective subscale.

The internal consistency of the subscales was assessed using post-test data with Cronbach’s Alpha and McDonald’s Omega. For positive self-compassion, values of 𝛼 = 0.752 and 𝜔 = 0.749 were obtained, while for negative self-compassion, both 𝛼 and 𝜔 were 0.715. These values fall within the acceptable range, exceeding the 0.70 threshold for internal consistency^51^.

#### Back-knowledge assessment

Back-related knowledge was assessed using a test consisting of nine questions, covering back-related content presented in the program’s educational videos. Each correct answer was awarded one point, and the individual scores were summed to generate a total score, ranging from 0 to 9. The questions addressed the following topics: the number of vertebrae, the mechanism of intervertebral disc nutrition, the shape of the spine, actions to improve posture, selection of a healthy spinal shape, disc loading during different activities, basic rules for lifting and carrying, the correct way to carry a schoolbag, and appropriate sitting posture. The question formats included a mix of single-choice and multiple-choice items with short text or image-based options, as well as one sequencing task requiring participants to arrange elements in the correct order.

#### Assessment of parental background information

Additional background information was collected through a parental questionnaire at both testing sessions. This included a brief health history, past posture-related problems, physical activity and sitting behavior, average daily sitting time on weekdays, and the parents’ educational background. Parental education was assessed using a scale based on the ISCED 2011 framework^52^ ranging from 1 to 8. Due to conceptual challenges associated with this construct^53^, the higher of the two parental education levels was used to represent the socioeconomic status (SES). For the second testing session, a shortened version of the parental questionnaire was used, focusing solely on sitting and physical activity behavior, as well as back-related complaints.

### Procedure and design

The study followed a 2 (group) × 2 (time) factorial design and adhered to CONSORT guidelines for randomized controlled trials^54^. Testing sessions took place at the laboratories of the Institute of Sport Science. Children attended the sessions accompanied by a parent, who remained outside the child’s visual field to minimize distraction and external influence. While testing was underway, parents completed a questionnaire providing additional background information. Each session lasted approximately 60 to 90 min. Informed consent was obtained from both children and their parents prior to testing.

The posture examination was conducted first, followed by the postural endurance test. Before the motor tests, participants completed the self-compassion questionnaire and a brief warm-up. Motor testing was conducted by a second examiner in a separate room, beginning with the FMS, followed by the back knowledge questionnaire. Trunk flexor endurance was then assessed, followed by the self-concept questionnaire and, subsequently, trunk extensor endurance. The session concluded with the PSK-K^55^, a physical self-concept questionnaire used to explore relationships in the pretest data. At the end of the pretest, group allocation was determined by simple randomization using a draw from a box containing an equal number of lots for each group (100 per group), prepared by the study coordinator at the outset of the study. Therefore, both participants and examiners were blinded until the pretest session was completed. Group assignment and explanation of the program were provided by the same person who conducted the motor tests. Participants in the IG also received habit cards for use during the intervention.

The posttest was conducted within two weeks of program completion and followed the same structure, omitting the initial administrative procedures. The PSK-K questionnaire was not administered at posttest, as it was used only in the pretest. Upon completing the study, each child received a €15 voucher as compensation. After data collection, children in the CG were offered the habit cards and introduced to the intervention program, which they were invited to complete. Due to this organizational group difference at the end of the testing session, blinding the examiner was impossible for the post-test.

The study was conducted in accordance with the ethical principles of the Declaration of Helsinki and received approval from the Ethics Committee of the University of Regensburg (approval number: 23–3522-101). Written informed consent was obtained from each child and from one parent or legal guardian after they were fully informed about the study procedures. The study was prospectively preregistered on the Open Science Framework (OSF; https://osf.io/3t67m/?view_only=2cf0b6189281440d8302a01497521a0f) prior to data collection, where the study design, hypotheses, and planned analyses were documented. All deviations from the preregistered protocol are described in detail in the section *Deviations from preregistration* at the end oft he manuscript. In addition, the trial was retrospectively registered in the German Clinical Trials Register (DRKS; Trial No.: DRKS00036556) on April 1, 2025, in compliance with journal requirements.

### Statistical analysis

Statistical analyses were performed using IBM SPSS Statistics 29 (IBM Inc., Chicago, IL, USA). Descriptive statistics were used to calculate frequencies, means, standard deviations (SD), and ranges. Group differences were analyzed using the Chi-square test, Fisher’s exact test, Mann–Whitney U test, or t-test for independent samples, depending on the distribution. The Wilcoxon test was used to assess changes in the number of postural abnormalities within each group, while a Chi-square test evaluated differences in the one-month back pain prevalence between groups. Two-factorial ANOVAs were conducted for the dependent variables FMS total score, back knowledge, the two self-compassion subscales, self-concept, postural endurance, and daily sitting time (the last two exploratory), with test time as the within-subject factor and group as the between-subject factor. Assumptions of homoscedasticity and homogeneity of covariance matrices were tested using Levene’s and Box’s M tests, respectively, and were met. For the two trunk endurance variables, one-way ANOVAs were calculated using the difference between pre- and post-test scores, as the assumption of homoscedasticity was violated and two-factorial ANOVAs could therefore not be applied.

To examine the influence of sex and SES on intervention effects in an exploratory manner, one-way ANOVAs with these fixed factors were conducted for the difference scores of the dependent variables previously analyzed via ANOVA in the IG. SES was dichotomized via median split to allow categorical analysis. Binary logistic regression analysis was conducted to explore whether the interaction between sex and growth predicted improvements in the physical performance tests. Furthermore, exploratory analyses were undertaken in the intervention group to examine whether adherence moderated intervention effects. For this purpose, a composite adherence index was calculated by z-standardizing the number of entries in the habit tracker and the exercise log and averaging these scores. Participants were then divided into high- and low-adherence groups via a median split of this index. For FMS, Adherence × Time interactions were tested, whereas for trunk flexor and trunk extensor endurance, consistent with the primary analyses, one-way ANOVAs were conducted on change scores (post–pre). Finally, exploratory analyses were also conducted to examine potential age-specific effects. For this purpose, participants were stratified into three age cohorts (youngest, middle, oldest tertiles) based on date of birth. Age Cohort × Time interactions were tested for FMS, and one-way ANOVAs on change scores were conducted for trunk flexor and extensor endurance.

Bonferroni correction was applied separately within each hypothesis to control for multiple comparisons and to reduce the risk of false positives. This resulted in adjusted significance thresholds ranging from *p* =.05 to *p* =.0167. The specific threshold used for each hypothesis is reported in the results section. Outliers were defined as values falling outside the range of mean ± 3 SD.

## Results

### Demographic data

Demographic data for both groups are presented in Table 1. The only significant difference between the groups was found in parental educational level. However, in both groups, most parents held a bachelor’s degree or higher, corresponding to the top three categories of the classification. One parent did not provide information on educational level. For daily sitting time, the values of two participants were excluded, as the reported values exceeded 24 h per day.

**Table 1:** Group comparison of demographic data.

		IG	CG	*p*-values of group difference
**Age M (SD)**		8.52 (1.34)	8.67 (1.26)	.455^a^
**Sex n [%]**				.927^b^
Boys		38 [53.5%]	38 [54.3%]	
Girls		33 [46.5%]	32 [45.7%]	
Highest parental educational level n [%] *				**0.047** ^**c**^
Not specified		0 [0%]	1 [1.4%]	
Completion of 10th grade		0 [0%]	1 [1.4%]	
A-levels		4 [5.6%]	1 [1.4%]	
Vocational school		2 [2.8%]	0 [0%]	
Short master craftsman training		5 [7.0%]	0 [0%]	
Bachelor´s degree		9 [12.7%]	9 [12.9%]	
Master´s degree		36 [50.0%]	47 [67.1%]	
PhD		15 [22.4%]	11 [15.9%]	
**Active in sports clubs n [%]**	None One club More clubs	10 [14.1%] 38 [53.5%] 23 [32.4%]	17 [24.3%] 34 [48.6%] 19 [27.1%]	.313^b^
**Active in programs outside of clubs n [%]**		22 [31.0%]	17 [24.3%]	.300^b^
Daily sitting time in hours M (SD)**		7.05 (1.38)	7.42 (1.63)	.146^d^
**One week back pain prevalence pretest N [%]**		6 [8.5%]	4 [5.7%]	.745^c^
**One-month back pain prevalence pretest N [%]**		10 [14.1%]	5 [7.1%]	.275^c^
**Three-month back pain prevalence pretest N [%]**		12 [16.9%]	9 [12.9%]	.637^c^
**Acute back pain at pretest N [%]**		1 [1.4%]	3 [4.3%]	.366^c^
**Assessed postural abnormalities in pretest M (SD)**		0.97 (1.29)	0.89 (1.37)	.530^a^

IG: *N* = 71; CG *N* = 70, * Not specified excluded in CG, ** Two outliers excluded in IG. a.

Mann-Whitney-U Test. b. Chi-Square Test. c. Fisher-exact Test. d. T-Test.

### Descriptive results

Table 2 presents the number of participants exhibiting at least one abnormality across different categories. A detailed breakdown of these categories, the frequency of each abnormality, and a more detailed description of the assessment process can be found in Supplementary Tables S2.1 and S2.2. In both groups, visual abnormalities related to the upper body and spine had the highest prevalence, with approximately one-third of participants displaying at least one abnormality in these areas.

**Table 2:** Frequency of postural abnormalities in different categories.

Summarized postural concept	IG		CG	
	Pretest *n* [%]	Posttest *n* [%]	Pretest *n* [%]	Posttest *n* [%]
Visual upper body and spine abnormalities	30 [42.3%]	22 [31.0%]	24 [34.4%]	23 [32.9%]
Visual lower extremity abnormalities	9 [12.7%]	10 [14.1%]	7 [10.0%]	15 [21.4%]
Functional restrictions	10 [14.1%]	11 [15.5%]	8 [11.4%]	6 [8.6%]

The mean values and standard deviations of the other measured variables can be found in Table 3 for both groups and test times.

**Table 3:** Overview of the remaining dependent variables, categorized by group and time point.

	IG		CG	
	Pretest M (SD)	Posttest M (SD)	Pretest M (SD)	Posttest M (SD)
Matthiass-test [s]	84.46 (28.52)	92.35 (30.13)	86.89 (30.15)	95.59 (31.54)
FMS	12.82 (3.04)	14.63 (2.90)	13.33 (3.24)	14.33 (3.41)
Trunk flexor endurance [s]	46.17 (39.47)	55.44 (39.56)	54.76 (47.20)	74.01 (76.73)
Difference from pre- to posttest [s]	9.27 (42.56)		19.26 (56.69)	
Trunk extensor endurance [s]	48.28 (33.76)	61.07 (36.97)	62.33 (39.09)	73.46 (49.39)
Difference from pre- to posttest [s]	12.79 (33.61)		11.13 (40.28)	
Back-knowledge	3.08 (1.35)	5.21 (1.58)	2.90 (1.45)	3.33 (1.55)
SCS-C_Positive self−compassion_	3.35 (0.70)	3.44 (0.75)	3.45 (0.78)	3.25 (0.81)
SCS-C_Negative self−compassion_	3.23 (0.82)	3.37 (0.79)	3.44 (0.74)	3.54 (0.73)
BSCI-Y	42.23 (10.02)	43.03 (9.93)	44.09 (7.66)	43.29 (7.87)

Participants in the intervention group completed the activity log on an average of 43 days (M = 42.39, SD = 28.00, range: 0–113) and recorded the frequency of their habit tasks on an average of 28 days (M = 27.51, SD = 24.81, range: 0–81). Values exceeding the 12-week program duration resulted from delays in completing the videos, leading to a delayed progression to the next program week. A total of 21 children from the intervention group completed at least one entry in both the activity log and the habit tracker per designated program week, indicating limited consistent engagement with these additional components.

### Postural changes and pain outcomes

**Fig. 2:**
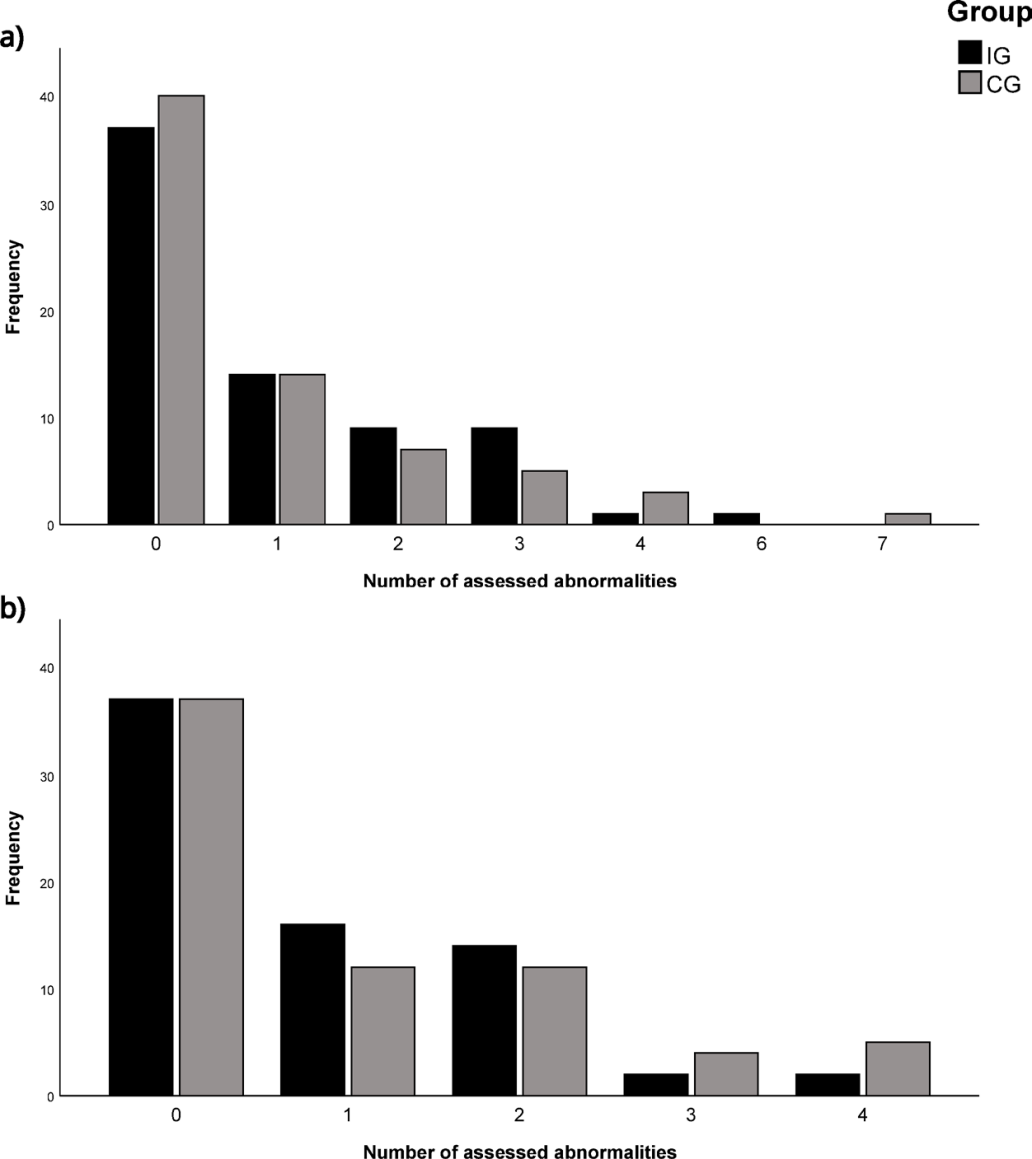
Number of assessed postural abnormalities: (a) Frequency at baseline; (b) Frequency at posttest.

Figure 2 illustrates the distribution of the assessed number of postural abnormalities across both groups and time points. In both groups, around half of the participants were asymptomatic (IG: 37 pre and post (52.1%), CG: 40 pre (57.1%) and 37 post (52.9%)). The frequency of individuals decreased as the number of assessed abnormalities increased. No substantial differences in distribution are observed between the groups. This finding is supported by the results of the Wilcoxon test at the corrected significance level *p* =.025, which indicates no significant change in the number of postural abnormalities per participant for both groups (IG: z = −0.858, *p* =.391, *r* = −.102; CG: z = 0.708, *p* =.479, *r* =.085). (Place Fig. 2 around here).

In the pre-test, 15 parents (10.6%; 10 IG, 5 CG) reported that their child had experienced back pain within the past month. By the post-test, this number had decreased to 8 (5.7%; 4 IG, 4 CG). Four participants (2 IG, 2 CG) reported back pain at both time points, while another four (2 IG, 2 CG) reported back pain only at the post-test. Consequently, eight participants in the intervention group no longer reported back pain after the program, compared to three participants in the control group. However, the change in one-month back pain prevalence did not differ significantly between the groups (χ²(1, 141) = 2.389, *p* =.122, φ = 0.130).

### Effects on core endurance and functional mobility

To account for multiple testing in the second hypothesis, the significance level was adjusted to *p* =.0167. For FMS total score, two participants were excluded as outliers, resulting in a final sample of N_IG_=71 and N_CG_=68. The analysis revealed no significant Group × Time interaction (F(1, 137) = 1.555, *p* =.214, partial η² = 0.011, 90% CI [0.000, 0.057]). A significant large main effect of Time was observed (F(1,137) = 42.837, *p* <.001, partial η² = 0.238, 90% CI [0.140, 0.332]), while the main effect of Group did not reach significance (F(1,137) = 0.356, *p* =.552, partial η² = 0.003, 90% CI [0.000, 0.034]). For trunk flexor endurance, four cases were identified as outliers and removed from the analysis, resulting in a final sample of N_IG_=70 and N_CG_=67. The change from pre- to post-test did not differ significantly between the two groups, F(1, 135) = 0.007, *p* =.936, η^2^ = 0.014, 90% CI [0.000, 0.004]. For trunk extensor endurance, three participants were excluded as outliers, leading to a final sample of N_IG_=71 and N_CG_=67. Similarly, the analysis did not reveal any significant pre-to-post differences between the groups, F(1, 136) = 0.641, *p* =.425, η^2^ = 0.052, 90% CI [0.000, 0.041]. Additional graphs visualizing these results can be found in Supplementary Figure S3; notably, the graphs for trunk muscle endurance suggest the presence of a time effect.

### Back-related knowledge

**Fig. 3:**
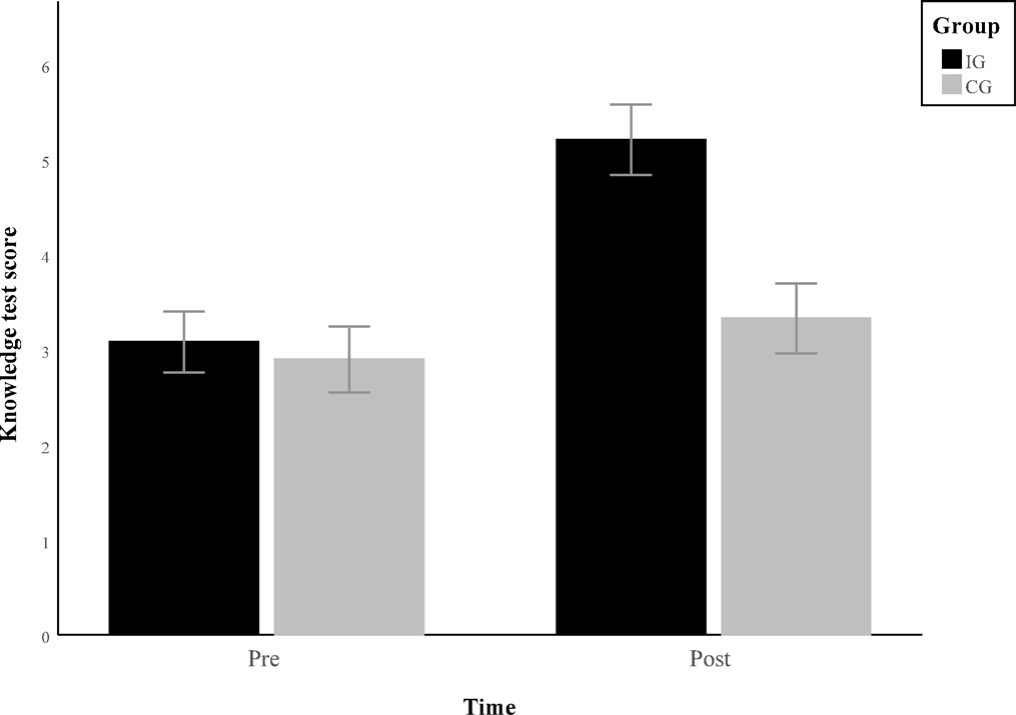
Group × Time interaction of knowledge test scores (mean ± 95% CI).

For the score in the knowledge test no correction of the significance level of *p* =.05 was necessary. Additionally, the data contained no extreme values. Figure 3 shows the significant interaction Group x Time (F(1, 139) = 40.955, *p* <.001, partial η^2^ = 0.228, 90% CI [0.132, 0.320]), which indicates a greater improvement of back-knowledge in the intervention group with a large effect size. (Place Fig. 3 here)

### Psychological effects

**Fig. 4:**
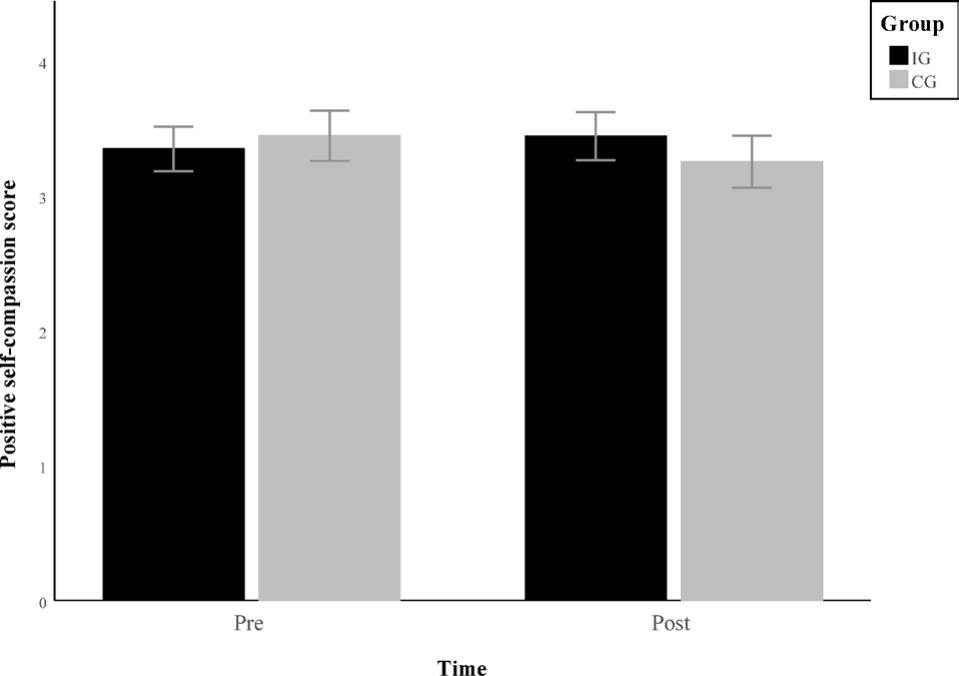
Group × Time interaction of positive self-compassion (mean ± 95% CI).

Since the psychological effects of the intervention were analyzed using three separate calculations, the significance level was adjusted to *p* =.0167 to account for multiple testing. For positive self-compassion, no outlier had to be excluded. The Group x Time interaction shown in Fig. 4 was significant (F(1, 139) = 6.560, *p* =.011, partial η² = 0.045, 90% CI [0.006, 0.112]) with a small to medium effect size. (Place Fig. 4 here)

The negative self-compassion was assessed in the sample N_IG_=N_CG_=70 excluding one extreme value from the IG. The analysis revealed no significant Group x Time interaction (F(1, 138) = 0.024, p =. 876, partial η² = 0.000, 90% CI [0.000, 0.013]). Neither the main effect of Time (F(1, 138) = 3.959, *p* =.049, partial η² = 0.028, 90% CI [0.000, 0.087]) nor for Group (F(1, 138) = 2.045, *p* =.155, partial η² = 0.015, 90% CI [0.000, 0.063]) reached significance after correction.

For self-concept, two cases from the IG were identified as outliers and removed from the analysis, resulting in the sample size N_IG_=69 and N_CG_=70. The Group × Time interaction (F(1,137) = 1.794, *p* =.183, partial η² = 0.013, 90% CI [0.000, 0.060]), as well as the main effects of Group (F(1,137) = 0.055, *p* =.814, partial η² = 0.000, 90% CI [0.000, 0.019]) and Time (F(1,137) = 0.009, *p* =.926, partial η² = 0.000, 90% CI [0.000, 0.006]) did not reach significance. The graphs illustrating the non-significant Group × Time interactions can be found in Supplementary Figure S3.

### Explorative analyses

#### Effects of the intervention on postural endurance and daily sitting time

For the Matthiass test, no outliers were present. The analysis revealed no significant Group × Time interaction (F(1,139) = 0.027, *p* =.869, partial η² = 0.000, 90% CI [0.000, 0.014]) or Group main effect (F(1, 139) = 0.407, *p* =.025, partial η² = 0.003, 90% CI [0.000, 0.035]). However, a significant main effect for Time was observed (F(1,139) = 11.393, *p* <.001, partial η² = 0.076, 90% CI [0.020, 0.0.153]), indicating a medium effect size. Three cases had to be excluded for the analysis of the daily sitting time as values above 24 h were reported, resulting in the sample N_IG_=N_CG_=69. The results show no significant effect for the Group x Time interaction (F(1, 136) = 0.059, *p* =.809, partial η² = 0.000, 90% CI [0.000, 0.020]), the Time (F(1, 136) = 0.148, *p* =.710, partial η² = 0.001, 90% CI [0.000, 0.027]) or Group main effect (F(1, 136) = 2.629, *p* =.107, partial η² = 0.019, 90% CI [0.000, 0.072]). The corresponding graphs for both analyses can be found in Supplementary Figure S4.

#### Influence of demographic factors and adherence

When exploring the influence of sex and SES as independent factors on the dependent variables FMS score, trunk flexor and extensor endurance, knowledge, self-compassion, and self-concept within the intervention group, only sex showed a significant effect. In contrast, no significant influence of SES was found. Sex had a large effect on the difference in trunk extensor endurance (F(1,67) = 13.738, *p* <.001, partial η² = 0.170, 90% CI [0.053, 0.299]). Boys (*N* = 38) had a mean difference of −0.16s (SD = 23.13), whereas girls (*N* = 33) showed a mean difference of 27.70s (SD = 37.74). A detailed overview of the test statistics, significance levels, and effect sizes for all analyzed dependent variables can be found in Supplementary Table S5.

Exploratory stratified analyses within the intervention group showed no consistent moderation of intervention effects by adherence. For FMS, a significant adherence × time interaction emerged, F(1,69) = 7.18, *p* =.009, partial η² = 0.094, 90% CI [0.013, 0.211], with the low-adherence subgroup showing greater improvement than the high-adherence subgroup. No significant interactions were observed for trunk flexor endurance, F(1,68) = 0.18, *p* =.673, partial η² = 0.003, 90% CI [0.000, 0.054], or trunk extensor endurance, F(1,69) = 0.06, *p* =.805, partial η² = 0.001, 90% CI [0.000, 0.038].

Exploratory analyses across three age cohorts (youngest, middle, oldest tertiles by age in months; *n* = 24, 24, and 23, respectively) did not reveal consistent age-specific intervention effects. For the FMS, the Age Cohort × Time interaction was not significant, F(2,68) = 1.94, *p* =.151, partial η² = 0.054, 90% CI [0.000, 0.143]. However, a main effect of age cohort emerged, F(2,68) = 3.89, *p* =.025, partial η² = 0.103, 90% CI [0.007, 0.209]. Post-hoc Tukey-HSD tests indicated that the youngest cohort (Pre: M = 14.38, SD = 3.37; Post: M = 15.42, SD = 2.87) scored significantly higher than the middle cohort (Pre: M = 12.00, SD = 2.55; Post: M = 13.92, SD = 3.16; *p* =.029), while differences between the youngest and the oldest cohort (*p* =.092) and between the middle and the oldest cohort (*p* =.889) were not significant. For trunk flexor endurance, one participant from the youngest cohort was excluded (consistent with the main analysis), resulting in *n* = 23, 24, and 23 per cohort. The ANOVA indicated no significant group differences, F(2,67) = 0.61, *p* =.548, partial η² = 0.018, 90% CI [0.000, 0.078], and Tukey-HSD tests confirmed that no pairwise contrasts reached significance (all *p* >.54). Similarly, for trunk extensor endurance (*n* = 24, 24, 23), group differences were not significant, F(2,68) = 0.37, *p* =.691, partial η² = 0.011, 90% CI [0.000, 0.058], with no pairwise contrasts significant (all *p* ≥.699).

#### Influences on physical improvements in the backfit program

To better understand potential factors within the intervention program that might have contributed to changes in back pain, exploratory subgroup analyses were conducted within the intervention group. Participants who showed improvements in back pain prevalence (*n* = 8) were compared to those who did not (*n* = 2), focusing on age, postural assessment outcomes, reported pre-existing conditions or known back problems, and physical performance data. However, no notable differences were identified between the two subgroups. A statistically significant difference could not be expected due to the small number of these groups in these exploratory analyses.

The significance levels of group differences from the exploratory comparisons between participants who showed improvements in the motor tests, specifically the two trunk endurance tests and the FMS, and those who did not are summarized in Table 4. A closer look at these subgroups revealed significant baseline differences across all three motor performance tests. For trunk flexor endurance (*p* <.001), the group with improvement had a mean baseline performance of 35.77 s (SD = 28.49), whereas those who did not improve averaged 66.54 s (SD = 49.65). For trunk extensor endurance (*p* =.038), the improvement group achieved a mean of 41.15 s (SD = 23.58), compared to 61.40 s (SD = 44.87) in the non-improvement group. In the functional mobility test, participants who did not improve had a baseline average of 14.26 points (SD = 3.03), while those who improved scored 12.13 points (SD = 2.82) in the pretest.

**Table 4:** Comparison of relevant factors between participants with and without improvements in the three physical performance tests.

Trunk flexor endurance		Improved (*n* = 47)			Not improved (*n* = 24)	
		*p*-values of group difference			Effect size	
Age		.920^a^			.01^a^	
Sex		.353^b^			.11^b^	
SES		.701^c^			.20^c^	
Growth		.607^a^			.06^a^	
Activity in sports club		.886^c^			.06^c^	
Baseline level		**< 0.001** ^**a**^			.43^a^	
**Trunk extensor endurance**		**Improved** **(n = 46)**			**Not improved** **(n = 25)**	
		**p-values of group difference**			**Effect size**	
Age		.479^a^			.08^a^	
Sex		.071^b^			.21^b^	
SES		.726^c^			.20^c^	
Growth		.400^a^			.10^a^	
Activity in sports club		.350^c^			.17^c^	
Baseline level		**0.038** ^**a**^			.25^a^	
**Functional mobility**		**Improved** **(n = 48)**			**Not improved** **(n = 23)**	
		**p-values of group difference**			**Effect size**	
Age		.455^a^			.09^a^	
Sex		.171^b^			.16^b^	
SES		.785^c^			.17^c^	
Growth		.755^a^			.04^a^	
Activity in sports club		.317^c^			.17^c^	
Baseline level		**0.005** ^**d**^			.74^d^	
	a		b	c		d
Used test	Mann-Whitney-U Test		Chi-Square Test	Fisher-exact Test		T-Test
Effect size	Pearson’s r		Cramer’s V	Cramer’s V		Cohen‘s d

The Growth factor represents the difference in height between posttest and pretest measurements.

To further explore the influence of sex-specific growth, interaction effects between sex and growth were tested using binary logistic regression for each of the three physical tests. In none of the models did the interaction term reach statistical significance (all *p* >.48). The interaction between sex and growth did not significantly predict improvement in the trunk flexor endurance, trunk extensor endurance, or the FMS score. The explained variance in all models was very low (Nagelkerke R² < 0.07), and the odds ratios for the interaction ranged between 1.25 and 1.41, indicating no meaningful effect.

## Discussion

This study examined the effects of a preventive digital back intervention designed to promote spinal health in children by targeting short-term improvements in posture, reducing back pain, and enhancing physical function, psychological well-being, and back-related knowledge. The findings present a nuanced picture: while the intervention significantly improved back-related knowledge, no group differences were observed for posture, back pain, trunk muscle endurance, functional mobility, or psychological outcomes. Each hypothesis is discussed in relation to the results and relevant literature in the following.

The observation of postural deviations in nearly half of the sample aligns with previous findings in children from the Czech Republic and a broader sample of children and adolescents from Brazil^3^. In contrast, the prevalence of back pain in this sample was relatively low at both time points. For context, a large-scale survey by the German health insurance provider DAK reported that 25% of 5th to 10th -grade students experienced back pain at least once per week—within a shorter reference period than the one-month prevalence assessed in the present study^56^. Similarly, a 2018 study found that 43% of German children and adolescents aged 0 to 17 reported experiencing back pain at least once per month^57^.

### Effects of the intervention

The first hypothesis must be rejected concerning improvements in posture and back pain prevalence. The distribution and change in postural abnormalities did not differ between groups or across test times. Similarly, the reduction in pain prevalence was not significantly different between groups. It is important to note that the posture assessment focused solely on rating detectable abnormalities without a usual medical examination, keeping the outer garments on and was not conducted using objective measurement tools. Subtle improvements over the three-month intervention period may therefore have gone unnoticed. Additionally, the posture observations were conducted by different research assistants with backgrounds or studies in medicine or physiotherapy. Although the assessors were studying in these fields, the lack of objective measurement, the potential variability in posture assessment, and the increased risk of errors must be acknowledged. In a separate comparison with rasterstereography and orthopedic examination focusing on individual postural parameters, discrepancies were found between methods^5^. The objective system tended to classify more children as having postural abnormalities, possibly due to stricter threshold values. Consequently, the results of the posture check should be interpreted with caution. Moreover, posture and its control in children are subject to diurnal and activity-related variation^58^ and can also be influenced by emotional states that fluctuate independently of the intervention^59^. Findings related to back pain should be interpreted with caution, as only a small number of participants reported symptoms at either time point. This limited prevalence particularly affected the exploratory subgroup analysis within the intervention group: due to the small number of cases, it was not possible to identify characteristics that might facilitate improvements in back pain as a result of the program. The absence of group differences in postural endurance improvements aligns with prior research in fifth-grade students^30^. A learning effect may explain the main effect of time. Additionally, increased motivation during the second testing session—regardless of group—may have improved performance. A ceiling effect could also affect results, as 31.2% of participants reached the 120-second maximum in the pre-test and 48.2% in the post-test.

The second hypothesis must also be rejected regarding changes in trunk endurance and functional mobility. One possible limitation was the difficulty children experienced in following exercise instructions via video. Gupta and Sehgal^60^ reported a generally high error rate when comparing video- to handout-based exercise instructions in children aged 10–12, citing ongoing cognitive development and lack of visual feedback, challenges likely even greater in our slightly younger sample. Similarly, difficulties were also observed during the physical performance tests despite prior demonstrations. Another factor that may have limited the effectiveness of the physical components of the intervention is the absence of mechanisms to verify whether exercises were performed correctly and with sufficient intensity. As the program was delivered remotely, children may not have always executed the movements as intended or may have engaged with the content only partially. Such variations in exercise fidelity and attentional engagement could have attenuated potential training effects and may partly explain the lack of significant improvements in trunk endurance and functional mobility. These challenges mirror the difficulties noted during the performance tests, where some children required repeated clarifications or failed to maintain the correct form even under direct supervision, suggesting that similar or greater inconsistencies likely occurred in the unsupervised home-based setting. Furthermore, primary school children generally engage in less intense physical activity than their older peers^61^, which may have limited the intervention’s effectiveness due to low training intensity. This effect could be amplified in home settings, as peers have been shown to increase physical activity in young children^62^. In addition, intrinsic motivation plays a key role in physical performance. It has been linked to higher test outcomes in children, whereas extrinsic motivators like verbal instruction or rewards at the testing show no such relationship^63^. Intrinsic motivation may vary across test times, which may have influenced the observed results. The significant main effect of time in the FMS may reflect either increased posttest motivation or a learning effect. A notable sex difference emerged when exploring trunk extensor endurance: average performance increased among girls but remained stable among boys. This is in line with a sex difference observed by Dejanovic et al.^64^, where girls outperformed boys across all ages from 7 to 14. As our exploratory observation is based on a small subgroup, future research should further examine the mechanisms behind these sex-specific patterns. In addition, given the realized sample sizes, post-hoc sensitivity analyses indicated that only small-to-moderate effects could be reliably detected, which may further explain the lack of significant group differences.

When exploring potential differences between participants who showed improvements in the motor tests and those who did not, results suggested that children with lower baseline scores were more likely to improve, indicating that those with weaker motor skills may particularly benefit from the exercise videos. Exploratory subgroup analyses further indicated that motor performance outcomes did not vary systematically by sex, SES, or recent growth status. Analyses across three age cohorts (youngest, middle, oldest tertiles based on date of birth) revealed no consistent age-specific intervention effects, with only a main effect of cohort for FMS but no interaction with time. Similarly, exploratory adherence analyses yielded no consistent moderation effects, although in the FMS the low-adherence subgroup showed greater improvement than the high-adherence subgroup. As all subgroup sizes were limited, these analyses remain exploratory and should be interpreted with caution.

The third hypothesis, regarding improvements in psychological well-being, has to be partially supported and partially rejected based on the mixed pattern of results. The missing effects are consistent with a meta-analysis that found no conclusive effects of physical activity interventions on mental health and well-being in children when adolescent samples were excluded^37^. It raises questions about the extent to which the body–mind connection, as suggested by embodiment theory^35^, is applicable in younger age groups. However, the improvement in positive self-compassion may be attributable to the structure of the exercise videos in the IG. Each video featured an adult, a child, and a mascot, fostering a warm and supportive atmosphere, especially during greetings and cooldowns. As self-compassion is closely associated with empathy and social connectedness^65^, this setting may have contributed to the observed group differences. In contrast, the CG watched brief, animated videos without human role models. Another possible explanation for this effect could be the simultaneous decrease in positive self-compassion in the control group, which, together with the increase observed in the intervention group, contributes to the observed interaction effect.

The fourth hypothesis, concerning improvements in back-related knowledge, can be accepted. These findings suggest that the results of Dullien et al.^30^ can be extended to a younger population and to remotely delivered educational content outside traditional classroom settings. Given the growing use of the internet for health-related purposes among children and adolescents—with information-seeking being one of the most common activities^66^ — short digital videos appear to be an effective and accessible method for knowledge transfer in this age group. Interestingly, however, the gain in knowledge did not lead to behavioral changes in sitting habits, as no greater reduction in daily sitting time was observed in the intervention group compared to the control group.

The lack of a significant influence of SES is somewhat unexpected, as socioeconomic status in this age group has previously been associated with greater access to physical activity opportunities and fewer opportunities for sedentary use^67^, both of which could have influenced outcomes in this study. However, caution is warranted when interpreting this result, as the overall educational level in the sample was high. Due to a median split, even children with one parent holding a master’s degree were sometimes classified into the lower SES group.

A broader challenge was the uneven adherence to the program, as reflected in the considerable variation in activity logs and habit trackers entries. Despite the web-based format and engaging content, the innovative approach aimed at fostering participation—suggested by Hill & Keating^16^—did not achieve the desired level of engagement. This was evident in participants’ progression: while some completed the program in the intended 12 weeks, others required the full 15 weeks or dropped out due to delays. In total, 36 participants (20.34%) discontinued the program—twice the estimated 10% dropout rate. Most attrition was due to low engagement or difficulties consistently completing the intervention.

A factor to consider when interpreting the results is the relatively young and developmentally broad age range of the sample, which likely influenced engagement, motivation, and adherence. Children in this age group show substantial variability in physical and cognitive development, behavior, and motivation^68–71^ and often require supervision due to limited self-regulation and shorter attention spans^72^ and may have affected the quality of engagement and the consistent use of program components in this study. Additionally, the intensity and quality of participation in the training and educational videos could not be monitored and feedbacked in this remote setting^73^, which may have affected engagement and the consistent use of program components.

As previously mentioned, the observed time effects may reflect group-independent increases in motivation during the second testing session. These effects may also be attributable to the project’s design: since group assignment occurred after the initial testing, participants had already expressed interest in a back prevention program. Even those assigned to the CG may have become more aware of the topic, sought information independently, or made changes to movement and sitting habits outside the intervention.

### Practical implications

Several practical implications can be drawn from these findings. Future back-prevention programs may benefit from integrating educational videos, as these proved effective in improving back-related knowledge. Since no significant improvements were observed in physical or psychological outcomes, further research is needed to identify effective strategies for this age group. Active video games have demonstrated a range of health benefits^74,75^, suggesting that enhanced gamification could improve both adherence and outcomes. Additionally, stronger involvement of parents or community networks, shown to be effective in mental health promotion^76^ and obesity prevention^77^, could enhance program impact. Moreover, future digital back-prevention programs should integrate mechanisms to monitor exercise fidelity and engagement. Approaches such as integrated digital tracking, parental support, or real-time feedback systems could help ensure that exercises are performed correctly and with sufficient intensity, and that participants fully engage with the educational content. Incorporating such elements may strengthen the effectiveness of remote interventions and support more consistent improvements, especially in physical outcomes.

### Strengths and limitations

Strengths of the study include its controlled design, multidimensional outcome assessment, and the comprehensive BackFit website, which enabled independent implementation. Among the limitations is the relatively short program duration of three months, which may have been insufficient, particularly when interrupted by illness or holidays. An additional shortcoming of this study is the lack of a later follow-up assessment, which prevents conclusions about the long-term preventive effects of the BackFit program. The main limitation is the lack of objectivity in posture assessment, which could be improved by incorporating more advanced tools such as a posture scanning system^5^. This type of postural assessment is more prone to measurement errors and observational bias, is less objective, and also complicates the replicability of the study. This represents the primary limitation of the study, requiring that the results of the posture assessment be interpreted with caution. The young and highly developmentally heterogeneous target group also presents a main weakness of the manuscript: motivation may fluctuate between test sessions or due to age-related differences, potentially affecting scores independently of the intervention. Moreover, the wide age range and sex-specific cognitive, physical and motivational developmental differences within the sample add further complexity to interpreting the results. In addition, the entirely remote delivery of the intervention meant that there was no opportunity to verify whether exercises were executed correctly, whether they were performed with sufficient intensity, or whether the educational videos were followed attentively. This lack of direct supervision constitutes a methodological limitation, as it may have particularly obscured potential physical improvements, which are highly dependent on accurate and intensive exercise execution. The target sample size determined by the a priori power analysis could not be achieved due to recruitment challenges. This may have reduced the statistical power to detect small to moderate effects and increased the likelihood of false negatives. Post-hoc sensitivity analyses conducted in G*Power^38^ indicated that, given the realized sample sizes and Bonferroni-adjusted α levels, only small-to-moderate effects could be detected reliably. This applies particularly to the physical outcomes, where detectable effect sizes were in the medium range. This suggests that smaller effects may have remained undetected. Full results for all hypotheses and outcomes are provided in the Supplementary Table S6. As sample sizes were especially insufficient for the conducted exploratory subgroup analyses, interpretations and suggestions based on these observations can only be considered assumptions and require further scientific investigation in larger samples. This sample limitation should be considered when interpreting the findings.

Lastly, the sample, characterized by high average SES and low back pain prevalence and considerable homogeneity in these areas, cannot be considered representative of the general population.

Taken together, these findings underscore the need for further refinement of digital back prevention programs for young children. Promising directions include stronger gamification, increased parental involvement, extended program duration, and tools to monitor participation and the consistency of program implementation more accurately.

## Conclusion

This study demonstrates that a web-based back prevention program can effectively enhance back-related knowledge among primary school children and improve positive self-compassion. However, no significant group differences were observed in physical or other psychological outcomes, and the use of the movement diary and habit tracker varied substantially between participants. These findings underscore the challenges of promoting health-related behaviors through digital interventions in young children, whose developmental stages and engagement levels can vary widely. Future programs may benefit from more interactive and motivating formats, greater involvement of caregivers, and improved tools to monitor participation and implementation fidelity. Additionally, the inclusion of more representative samples would improve the generalizability of findings. The large physical differences within the wide age range from 6 to 11 years needs to be considered for further investigation focussing on a smaller age range. Clinical examination must be considered according to medical requirements. A bigger number of participants is necessary to assess the performed physical testings.

### Deviations from preregistration

Several minor deviations from the preregistered protocol occurred during the study. The manuscript title was revised to more accurately reflect the study design and focus. The structure of the hypotheses was adjusted to group related outcomes and improve clarity, which also led to the application of different significance thresholds due to corrections for multiple comparisons. One hypothesis, originally formulated to examine a general time effect in the postural endurance test, was reclassified as part of the exploratory analyses, as the confirmatory analyses focused on outcomes for which greater improvements were expected in the intervention group. Due to inconsistent use of the daily movement diary and habit tracker, only the average number of days with completed entries was reported. Exploratory subgroup analyses were also conducted to investigate potential differences in program responsiveness among various subgroups of the sample. As described in the statistical analysis section, one-way ANOVAs were used for the trunk endurance variables, as the assumptions required for two-way ANOVAs were not met. Furthermore, the calculated sample size could not be achieved due to external constraints, as detailed in the Methods section.

## Supplementary Information

Below is the link to the electronic supplementary material.

Supplementary Material 1

Supplementary Material 2

## Data Availability

All data generated or analyzed during this study are included in this published article and its supplementary information files.
