# Supplementary material for: Evaluation of a web-based back prevention program for primary school children: a randomized controlled trial
Source: Sci Rep. 2025 Nov 21;15:41176. doi: 10.1038/s41598-025-27813-0 (PMC12638964; doi:10.1038/s41598-025-27813-0)
Supplement: Supplementary file 2 — Supplementary Material 2 [file 41598_2025_27813_MOESM2_ESM.pdf]

a)

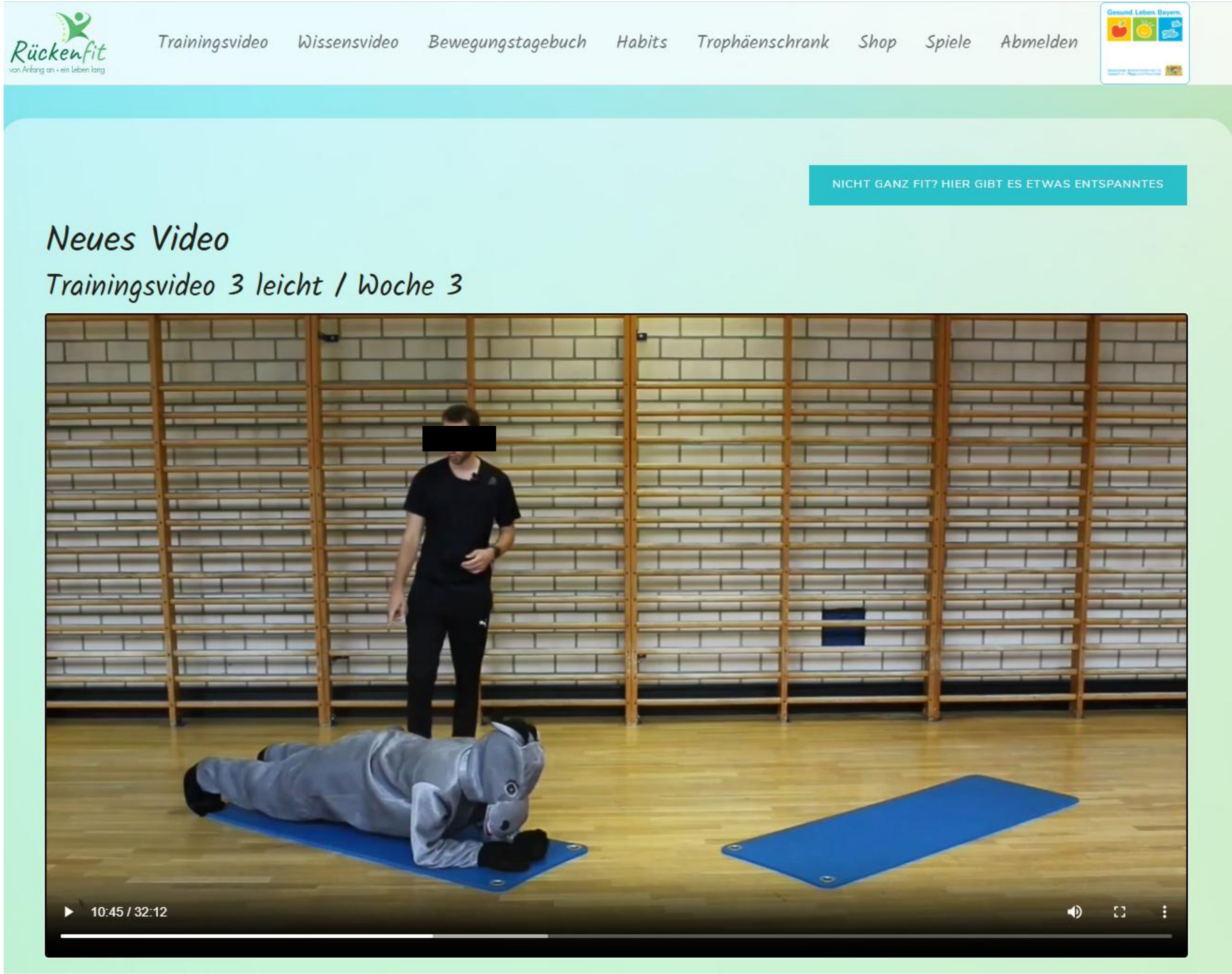
Rückenfit
von Anfang an • ein Leben lang
Trainingsvideo
Wissensvideo
Bewegungstagebuch
Habits
Trophäenschrank
Shop
Spiele
Abmelden
Gesund. Leben. Bayern.
NICHT GANZ FIT? HIER GIBT ES ETWAS ENTSPANNTES
Neues Video
Trainingsvideo 3 leicht / Woche 3
10:45 / 32:12

b)

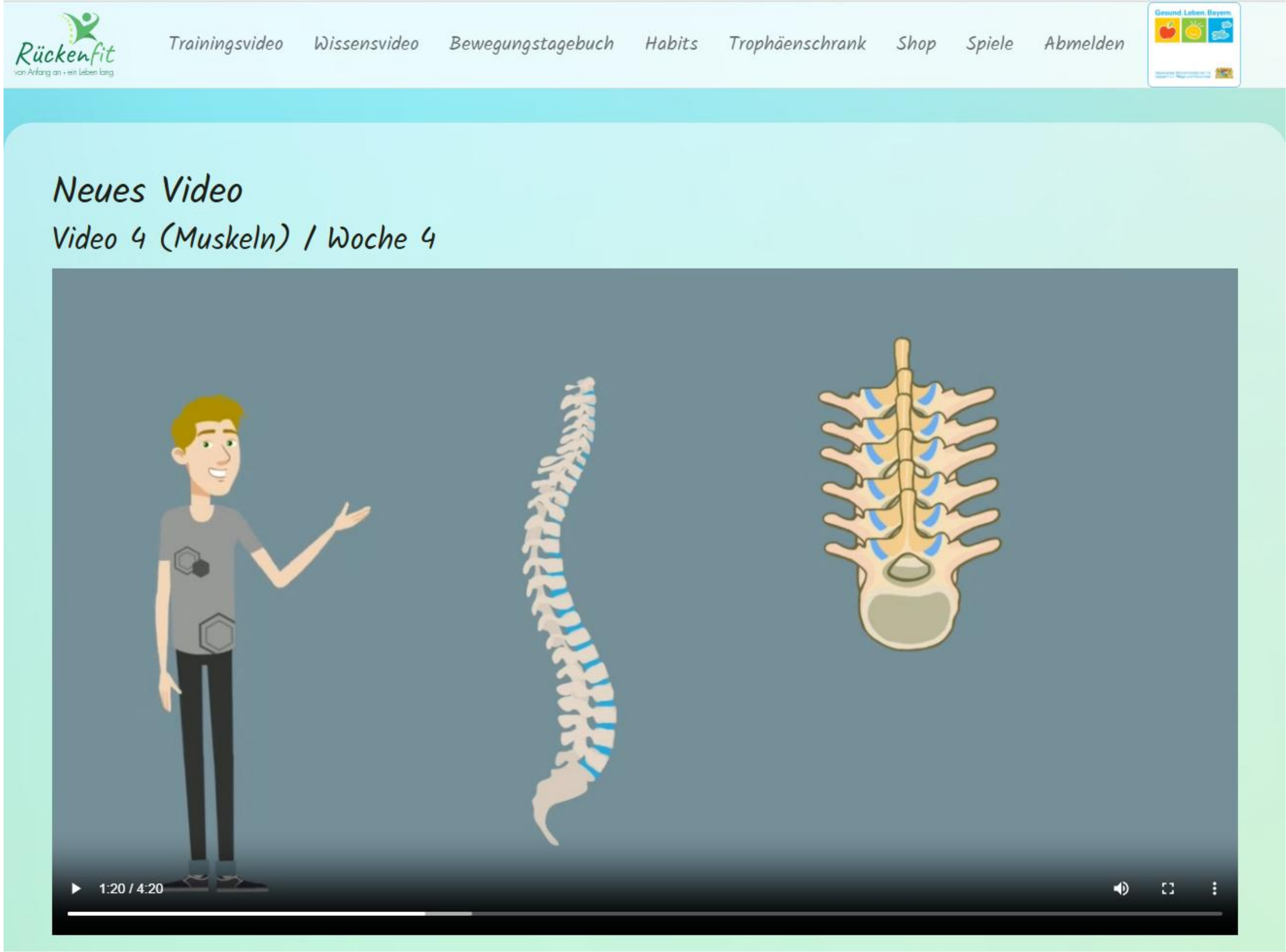
Rückenfit
von Anfang an • ein Leben lang
Trainingsvideo
Wissensvideo
Bewegungstagebuch
Habits
Trophäenschrank
Shop
Spiele
Abmelden
Gesund. Leben. Bayern.
Neues Video
Video 4 (Muskeln) / Woche 4
1:20 / 4:20

c)

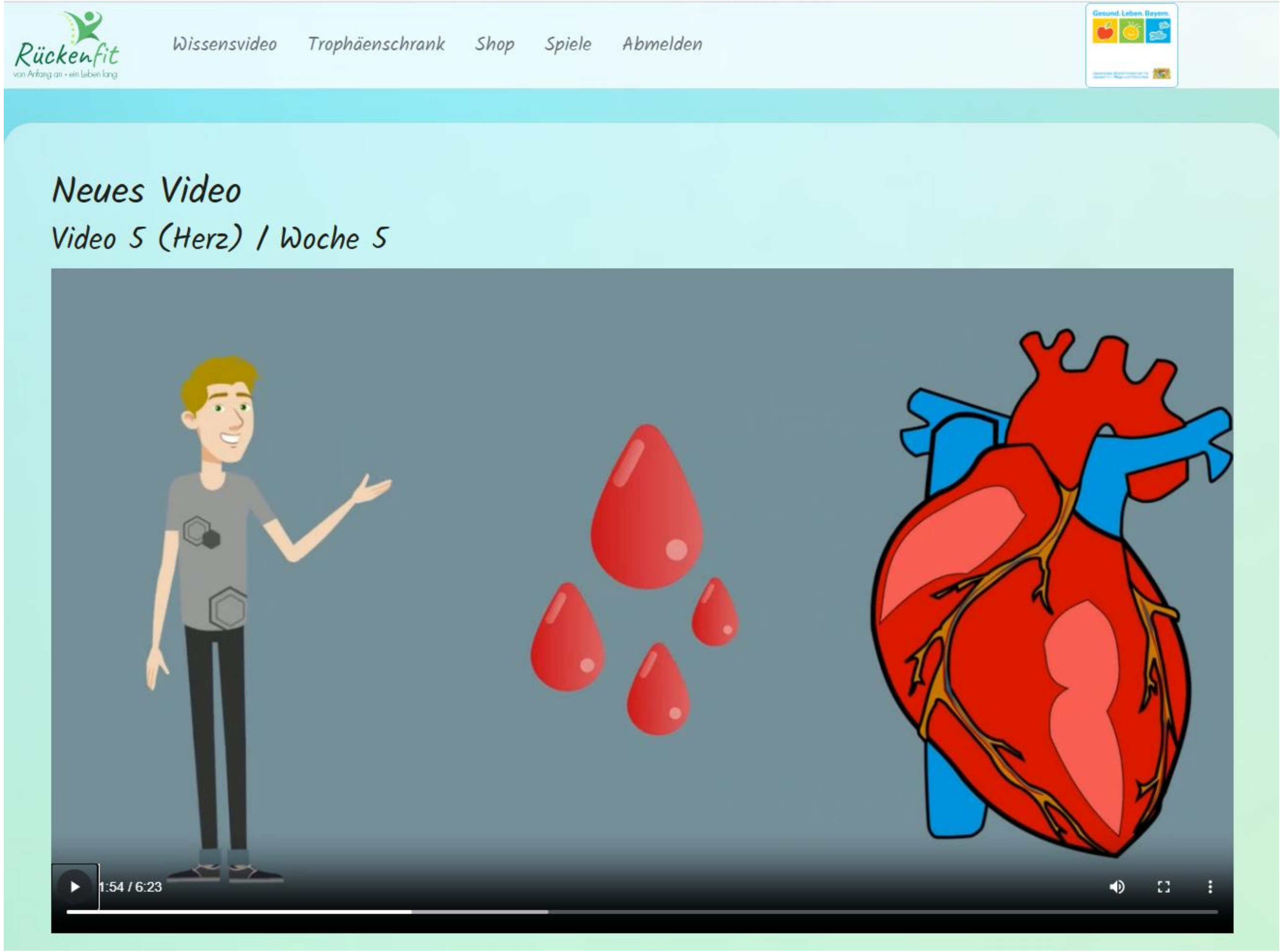

Supplementary Figure S1: The Backfit Website: a) IG Exercise Video; b) IG Knowledge Video; c) CG Knowledge Video

Classification of the assessed postural abnormalities

Summarized concept	Included abnormalities	IG		CG	
		Pretest n [%]	Posttest n [%]	Pretest n [%]	Posttest n [%]
Visual upper body and spine abnormalities	Shoulder tilt	3 [4.2%]	6 [8.5%]	4 [5.7%]	7 [10.0%]
	Plumb line deviation	2 [2.8%]	0 [0%]	1 [1.4%]	0 [0%]
	Cervical lordosis	6 [8.5%]	7 [9.9%]	9 [12.9%]	4 [5.7%]
	Thoracic kyphosis	11 [15.5%]	8 [11.3%]	13 [18.6%]	12 [17.1%]
	Lumbar lordosis	19 [26.8%]	5 [7.0%]	15 [21.4%]	6 [8.6%]
Visual lower extremity abnormalities	Leg axis	9 [12,7%]	4 [5.6%]	4 [5.7%]	2 [2.9%]
	Foot position	1 [1.4%]	5 [7.0%]	3 [4.3]	7 [10.0%]
	Knee axis	1 [1.4%]	3 [4.2%]	0 [0%]	10 [14.3%]
Functional restrictions	Gait pattern	2 [2.8%]	0 [0%]	0 [0%]	0 [0%]
	Single leg-stance	0 [0%]	0 [0%]	0 [0%]	0 [0%]
	Toe walking	0 [0%]	0 [0%]	0 [0%]	0 [0%]
	Heel walking	0 [0%]	0 [0%]	0 [0%]	0 [0%]
	Spinal rotation	6 [8.5%]	6 [8.5%]	7 [10.0%]	2 [2.9%]
	Spinal reclination	5 [7.0%]	6 [8.5%]	3 [4.3%]	5 [7.1%]

Supplementary Table S2.1: Classification of the Assessed Postural Abnormalities

Note. Only the standardized abnormalities from the postural assessment were categorized, not the additional observations.

Assessment of the postural abnormalities

Postural abnormalities	Assessment process
Shoulder tilt	A shoulder elevation compared to the opposite side when viewed from the front was assessed, with clearly visible asymmetry classified as an abnormal finding.
Plumb line deviation	Vertical alignment was assessed from the frontal view for lateral deviation (shift), defined as a sideways displacement of the spine with the shoulder girdle translated to one side relative to the pelvis.
Cervical lordosis	From the lateral view, cervical spine alignment was assessed for lateral head shift (side deviation of the head relative to the shoulder girdle, using chin and sternum as reference points) and for protraction, defined as the head being positioned forward rather than aligned over the shoulders and pelvis (“turtle neck”).

Thoracic kyphosis	Thoracic spine posture was assessed from the lateral view for hyperkyphosis, indicated by very laterally positioned shoulder blades, and hypokyphosis, defined as an absence of visible thoracic curvature.
Lumbar lordosis	Lumbar spine posture was assessed from the lateral view for hyperlordosis, characterized by anterior pelvic tilt, posterior displacement of the thorax, and forward abdominal protrusion, and for hypolordosis, defined as a lack of curvature with a flat transition between the sacrum and thoracic spine.
Leg axis	The child was asked to stand with the legs touching, aiming for alignment of the anterior superior iliac spines, patellae, and medial malleoli in a straight line; an abnormal finding was noted if the knees touched but the ankles did not, or vice versa.
Foot position	Foot posture was assessed for high arches (increased longitudinal arch), flat feet (flattened longitudinal arch, physiological up to age 10), and valgus heel position (pronation). As flat feet and valgus positions often occur together, a single-heel-rise test was performed; if the foot alignment corrected during this test, the valgus position was considered muscular and not classified as an abnormal finding.
Knee axis	Knee alignment was assessed from the lateral view, with hyperextension of around 10 degrees considered physiological and hyperextension exceeding approximately 15 degrees classified as abnormal, while an extension deficit (less than 0 degrees) was noted as unphysiological and a potential indicator of leg length discrepancy.
Gait pattern	Gait was assessed as the participant walked a distance of approximately 5 meters away from and back towards the examiner, screening for Trendelenburg gait and steppage gait patterns.
Single leg-stance	Single-leg stance was assessed by asking the participant to maintain balance on one leg for approximately three seconds.
Toe walking	Toe walking was assessed by asking the participant to stand and maintain balance on their toes for approximately three seconds.
Heel walking	Heel walking was assessed by asking the participant to stand and maintain balance on their heels for approximately three seconds.
Spinal rotation	Spinal rotation was assessed in a seated position with the child's feet on the floor and knees stabilized against a wall to prevent hip or knee movement, measuring active rotation to both sides. Marked limitations, defined as rotation of noticeably less than approximately 40 degrees, were recorded as abnormal findings.
Spinal reclination	Spinal extension was assessed by asking the child to place their hands on their hips and extend the spine backwards while standing without assistance. Limitations with extension of noticeably less than approximately 60 degrees were recorded as abnormal findings.

Supplementary Table S2.2: Assessment Process of Postural Abnormalities

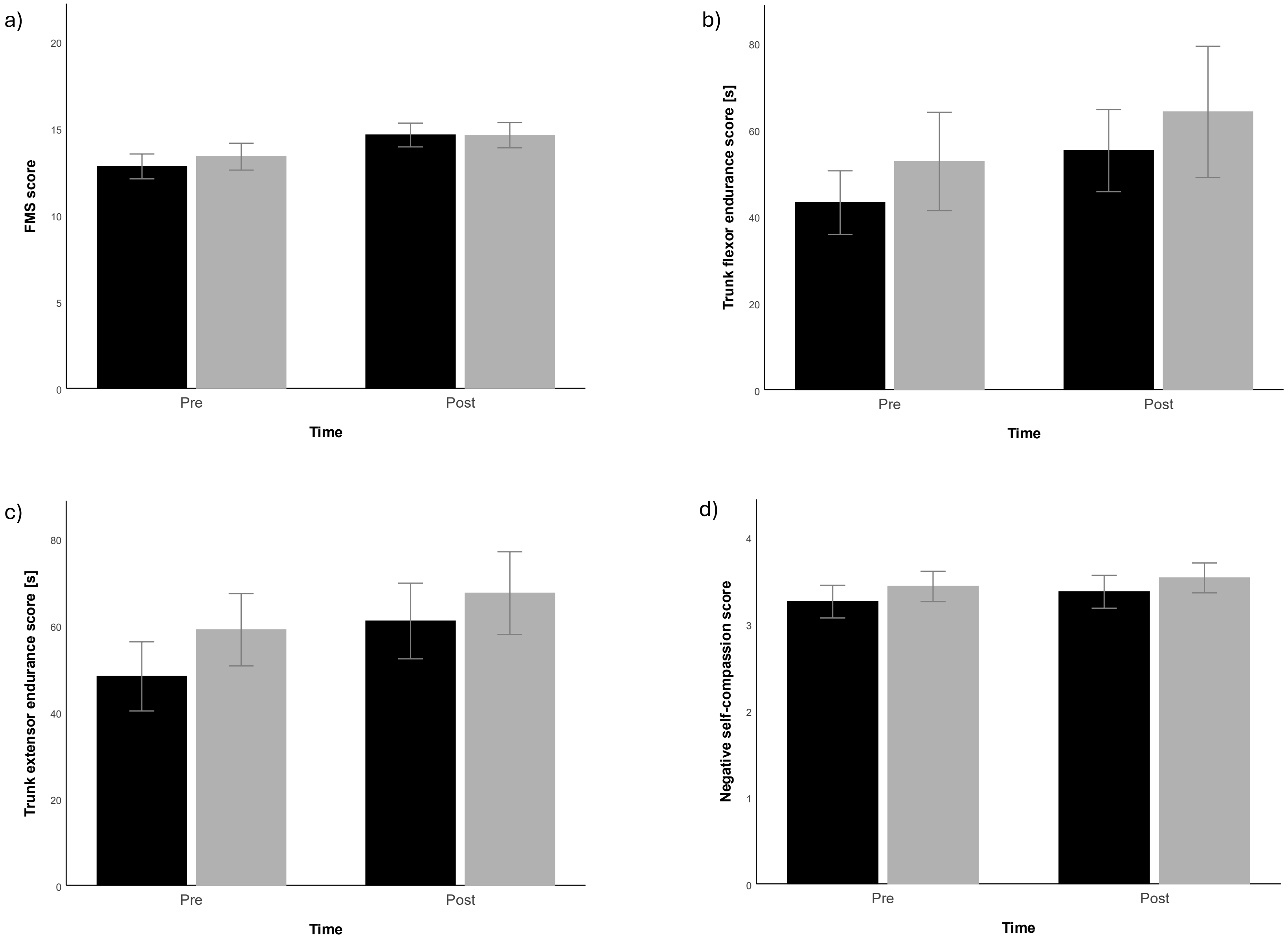

a)
FMS score
0
5
10
15
20
Pre
Post
Time
b)
Trunk flexor endurance score [s]
0
20
40
60
80
Pre
Post
Time
c)
Trunk extensor endurance score [s]
0
20
40
60
80
Pre
Post
Time
d)
Negative self-compassion score
0
1
2
3
4
Pre
Post
Time

e)

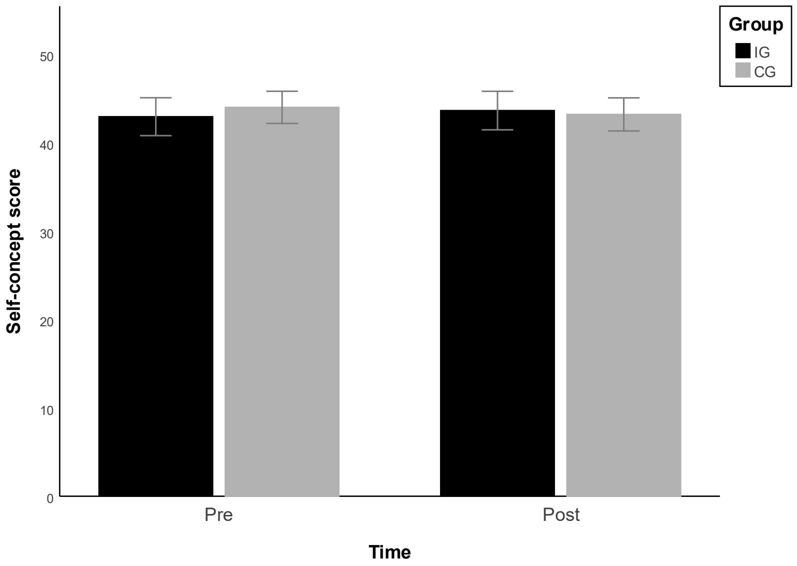

Supplementary Figure S3: Visualization of estimated marginal means of a) FMS, b) Trunk flexor endurance, c) Trunk extensor endurance, d) Negative self-compassion, e) Self-concept

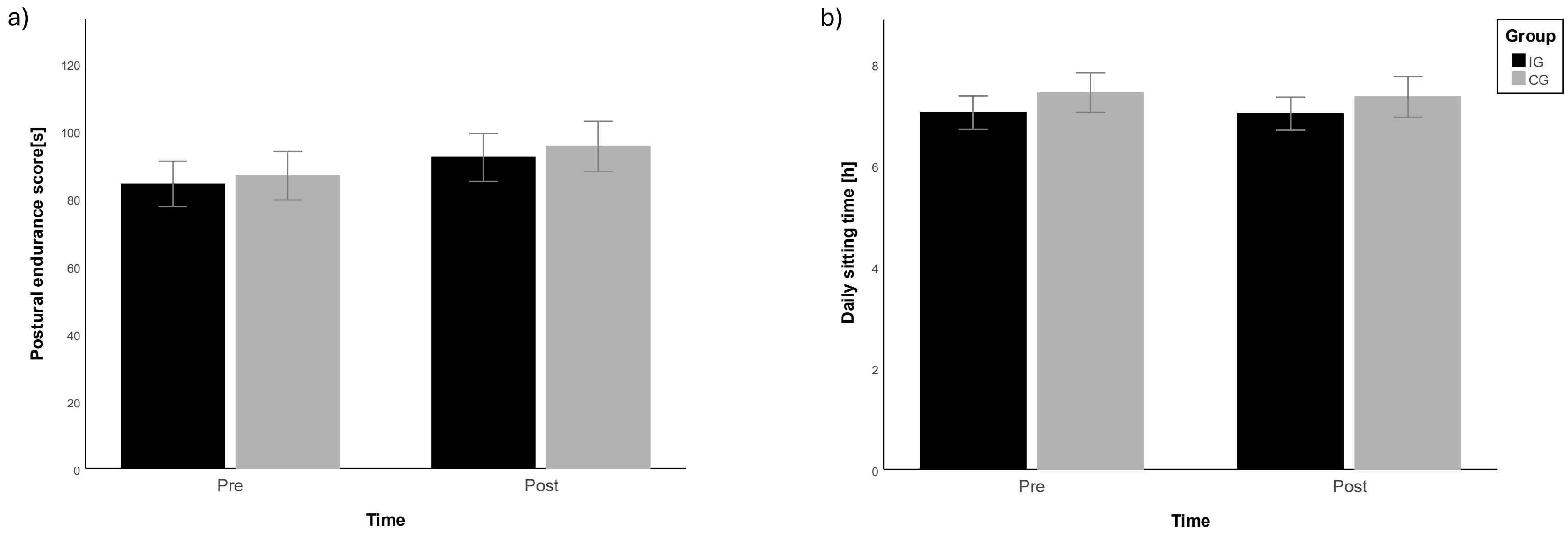

Additional Figure S4: Visualization of estimated marginal means of a) Postural endurance, b) Daily sitting time

FMS

	Test statistics	p-value	Partial η²
Sex	F(1, 67)=1.907	.172	.349
SES	F(1, 67)=0.667	.417	.010
Sex x SES	F(1, 67)=0.352	.555	.005

Trunk flexor endurance

	Test statistics	p-value	Partial η²
Sex	F(1, 66)=0.828	.366	.012
SES	F(1, 66)=1.902	.172	.028
Sex x SES	F(1, 66)=0.14	.905	.000

Trunk extensor endurance

	Test statistics	p-value	Partial η²
Sex	F(1, 67)=13.738	< .001	.170
SES	F(1, 67)=0.019	.890	.000
Sex x SES	F(1, 67)=0.608	.438	.009

Knowledge test

	Test statistics	p-value	Partial η²
Sex	F(1, 67)=0.145	.705	.002
SES	F(1, 67)=2.250	.138	.032
Sex x SES	F(1, 67)=0.089	.767	.001

Self-compassion

	Test statistics	p-value	Partial η²
Sex	F(1, 67)=0.050	.823	.001
SES	F(1, 67)=0.124	.726	.002
Sex x SES	F(1, 67)=0.042	.838	.001

Self-concept

	Test statistics	p-value	Partial η²
Sex	F(1, 67)=0.678	.413	.010
SES	F(1, 67)=0.025	.875	.000
Sex x SES	F(1, 67)=0.382	.539	.006

Postural endurance

	Test statistics	p-value	Partial η²
Sex	F(1, 67)=0.058	.811	.001
SES	F(1, 67)=0.157	.693	.002
Sex x SES	F(1, 67)=0.042	.839	.001

Supplementary Table S5: Influence of Sex and SES

Post-hoc Sensitivity Analyses by Hypothesis and Outcome

**Hypothesis**	**Outcome**	**Significance level**	***N* =**	**Repeated measures *r***	**MDE**
H1	Posture	α = .025	71 (IG)<br>70 (CG)	—<br>—	*dz* = .38<br>*dz* = .38
H1	Back pain prevalence	α = .025	141	—	*w* = .26
H2	Functional Mobility	α = .0167	139	.30 /.50 / .70	partial η^2= .026 / .019 / .011
H2	Trunk flexor endurance	α = .0167	137	—	*d* = .55
H2	Trunk extensor endurance	α = .0167	138	—	*d* = .55
H3	Positive self-compassion	α = .0167	141	.30 /.50 / .70	partial η^2= .026 / .019 / .011
H3	Negative self-compassion	α = .0167	140	.30 /.50 / .70	partial η^2= .026 / .019 / .011
H3	Self-concept	α = .0167	139	.30 /.50 / .70	partial η^2= .026 / .019 / .011
H4	Back-knowledge	α = .05	141	.30 /.50 / .70	partial η^2= .020 / .014 / .008

Supplementary Table S6: Post-hoc sensitivity analysis

Note. MDE = minimum detectable effect. MDEs are reported as partial η^2 for repeated measures ANOVAs, Cohen's *d* for independent-samples t-tests, *dz* for paired-samples tests, and Cohen's *w* for χ^2-tests.
